# The epigenetic landscape of clear-cell renal cell carcinoma

**DOI:** 10.15586/jkcvhl.2015.33

**Published:** 2015-05-28

**Authors:** Katarzyna Kluzek, Hans A Bluyssen, Joanna Wesoly

**Affiliations:** 1 Department of Human Molecular Genetics, 2 Laboratory of High Throughput Technologies, Institute of Molecular Biology and Biotechnology, Faculty of Biology, Adam Mickiewicz University in Poznan, Umultowska 89, 61-614 Poznan, Poland.

## Abstract

Clear cell renal cell carcinoma (ccRCC) is the most common subtype of all kidney tumors. During the last few years, epigenetics has emerged as an important mechanism in ccRCC pathogenesis. Recent reports, involving large-scale methylation and sequencing analyses, have identified genes frequently inactivated by promoter methylation and recurrent mutations in genes encoding chromatin regulatory proteins. Interestingly, three of detected genes (PBRM1, SETD2 and BAP1) are located on chromosome 3p, near the VHL gene, inactivated in over 80% ccRCC cases. This suggests that 3p alterations are an essential part of ccRCC pathogenesis. Moreover, most of the proteins encoded by these genes cooperate in histone H3 modifications. The aim of this review is to summarize the latest discoveries shedding light on deregulation of chromatin machinery in ccRCC. Newly described ccRCC-specific epigenetic alterations could potentially serve as novel diagnostic and prognostic biomarkers and become an object of novel therapeutic strategies.

## Introduction

Kidney cancer is one of the 15 most common malignancies occurring globally, with more than 270,000 new cases every year worldwide (1–3). The majority of malignant kidney tumors are renal cell carcinomas (RCC) with the most common and aggressive subtype being clear-cell renal cell carcinoma (ccRCC), comprising approximately 70% of all kidney tumors (4). Localized ccRCC is potentially curable by resection, though about 30% of patients relapse after initial nephrectomy (5). Unfortunately, ccRCC is frequently non-symptomatic in the early phases, and is repeatedly detected in advanced stage often with metastases (6). When metastasized, ccRCC is chemo- and radiation-resistant and in most cases remains incurable, resulting in a 95% mortality rate (7, 8).

To date no effective ccRCC treatment has been developed and none of the potential biomarkers have been approved for clinical application. For many years von Hippel-Lindau (VHL) tumor suppressor gene (TSG) was the only TSG associated with ccRCC pathogenesis (9). Attempts to detect other mutated genes have been unsuccessful for a long time, though deregulation of chromatin machinery has recently emerged as an important mechanism in renal neoplasms. Large-scale sequencing projects have identified novel TSGs, mapped to the frequently lost 3p21 locus and functioning as epigenetic chromatin and/or histone modifiers, indicating epigenetic changes may play an important role in ccRCC development (10–12). Silencing of VHL through promoter methylation in ccRCC was one of the first examples of this phenomenon and so far approximately 60 genes have been suggested to be epigenetically deregulated in ccRCC (13). Here, we summarize the most recent discoveries in the field of ccRCC epigenomics, providing potential diagnostic and prognostic biomarkers as well as possible novel targets for therapeutic intervention.

## Epigenetic alterations in ccRCC

The main mechanisms responsible for chromatin state regulation are: DNA methylation, nucleosome remodeling, and covalent histone modifications through methylation, acetylation, phosphorylation, ubiquitination, or sumoylation. These modifications can directly change DNA organization and/or accessibility as well as lead to the recruitment of proteins altering chromatin structure and in consequence influence transcription, replication, recombination and DNA repair (14, 15). Recent genome-wide methylation studies and sequencing projects demonstrated that the disruption of epigenetic control has a significant role in the initiation and progression of ccRCC (16–18).

### Inactivation of potential tumor suppressor genes through DNA methylation

DNA methylation is the best studied epigenetic modification and the only epigenetic mark with a well described mechanism of mitotic inheritance (19). It plays an important role in various biological processes, for example, genomic imprinting, transposable elements silencing, and embryonic development (20). Methylation patterns are generated and maintained by DNA methyltransferases (DNMTs). DNMT1 acts during replication and maintains methylation of the new DNA strand, DNMT3a and DNMT3b are de novo methyltransferases that act independently of replication and display no preference for unmethylated nor hemi-methylated DNA (20–23).

The majority of CpG-rich promoter regions (CpG islands) occupying near 60% of human gene promoters usually remain unmethylated (24). Gene silencing by promoter region methylation of TSGs is a frequent mechanism described in human cancers, with epigenetic inactivation of VHL in ccRCC being one of the first examples (13, 25, 26). VHL, while mutated in approximately 80% of sporadic ccRCC, is inactivated by methylation in an additional ~10% of cases (27, 28). Identification of other epigenetically inactivated TSGs was an important approach to study the pathogenesis of ccRCC, and promoter hypermethylation of several genes commonly inactivated in ccRCC has been documented (18). Based on a search of online databases, compilation of candidate genes reported in numerous studies to show tumor-specific hypermethylation in ccRCC, has been published in 2010 (28). Morris et al. described 38 genes methylated in ccRCC, among those only a small number was methylated with high frequency (≥50% of cases: APAF1, COL1A1, DKK2, DKK3, SFRP2, SFRP4, SFRP5, and WIF1) while rarely (<10%) in matched normal tissue (28).

The earlier, initial studies mostly implemented targeted, candidate-driven analyses. Recently, several whole genome strategies also have been applied. A large functional epigenetic screen of gene upregulation post 5-aza-2’-deoxycytidine demethylation treatment by high-density gene expression microarrays in 11 RCC cell lines (KTCL 26, RCC4, UMRC2, UMRC3, SKRC18, SKRC39, SKRC45, SKRC47, SKRC54, 786-0 and Caki-1) was applied by Morris *et al*. Genes re-expressed after demethylation were validated in 61 primary tumors (~80% clear cell and 20% non-clear cell RCC). Five genes (BNC1, COL14A1, CST6, PDLIM4, and SFRP1) demonstrated frequent tumor-specific promoter region methylation (>30%), associated with transcriptional silencing. Re-expression of BNC1, CST6, and SFRP1 suppressed the growth of RCC cell lines, whereas RNAi knock-down of BNC1, SFRP1, and COL14A1 increased their growth, suggesting tumor suppressor activity (29). Similarly, methylated DNA immunoprecipitation (MeDIP) of primary tumors, followed by high-density whole-genome expression microarray comparative analysis revealed 9 genes frequently methylated in primary ccRCC tumour samples: PCDH8 (58%), KLHL35 (39%), ATP5G2 (36%), CCDC8 (35%), FBN2 (34%), ZSCAN18 (32%), their promoter hypermethylation resulting in gene silencing (30). None of these genes have been reported previously to be methylated in RCC nor other cancers.

Genome-wide DNA methylation studies in ccRCC have also been performed using BeadChip arrays. Comparison of DNA methylation profiles in familial (n = 29) and sporadic (n = 20) VHL^+/+^ ccRCC showed more frequently methylated RASSF1, PITX2, CDH13, HS3ST2, TWIST1, TAL1, TUSC3, and DCC loci in sporadic cases, indicating differences in tumorigenesis mechanisms dependent on VHL status (31). Several novel ccRCC TSG candidates (SLC34A2, OVOL1, DLEC1, TMPRSS2, SSTand BMP4) have been found in a global study of CpG methylation in 38 ccRCC and 9 age-matched healthy tissues (~27,500 CpGs and >14,000 genes) (32). All of those exhibited frequent transcriptional silencing associated with promoter methylation (20–60% of cases).

Dmitriev *et al*. focused on genetic and epigenetic destabilization of genes on chromosome 3 (33). The study (validated by bisulfite genomic sequencing) showed 22 genes displaying high frequency of methylation (17–57%) and/or deletion in ccRCC. Identified genes included well-known TSGs VHL, CTDSPL, LRRC3B, ALDH1L1, and EPHB1, but also genes not previously linked to cancer development (LRRN1, GORASP1, FGD5, and PLCL2). Proteins encoded by a part of these genes are involved in signaling pathways and biological processes frequently affected in cancer, like apoptosis (GORASP1), regulation of actin cytoskeleton (FGD5), transmembrane signaling systems (GNAI2) or regulation of NFkappaB activity (NKIRAS1). Dmitriev *et al*. further confirm that mechanism of ccRCC development is linked to destabilization of genes at chromosome 3, discussed in more detail in the next paragraph.

Studies described above have identified a large number of genes methylated in sporadic ccRCC. There is small overlap between studies and consensus on which genes play a role in its etiology and whether any of those are of relevance clinically. However, all of the reported genes are involved in processes often deregulated during tumorigenesis: apoptosis, proliferation, cell survival and tumor invasion. The Cancer Genome Axis (TCGA) Kidney Renal Clear Cell Carcinoma (KIRC) database provides an excellent opportunity to confirm and unify previously obtained results (16). These data include 199 ccRCC tumor/normal paired analyses using the Infinium HumanMethylation27 BeadChip validated on 160 ccRCC tumor/normal paired samples using the Infinium HumanMethylation450 BeadChip.

### Mutations of genes regulating epigenetic modifications

**Figure 1.**
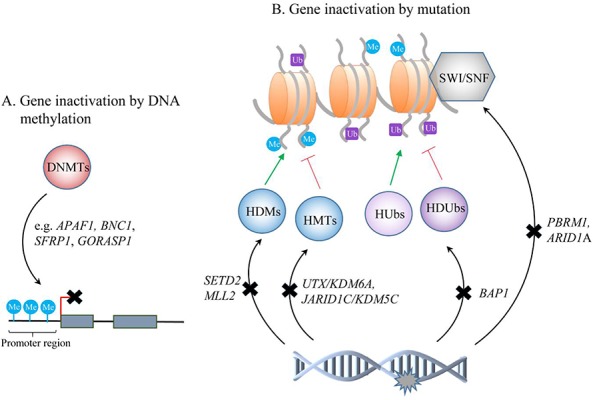
Schematic representation of epigenetic changes identified in ccRCC tumors. DNMTs - DNA methyltransferases; HDMs - histone demethylases; HMTs - histone methyltransferases; Hubs - histone ubiquitinases; HDUbs - histone deubiquitinases; SWI/SNF - chromatin remodeling complex.

Non-covalent mechanisms, such as nucleosome remodeling can change chromatin structure and influence gene activity by altering the accessibility of regulatory DNA sequences to transcription factors (34). Currently, there are four known families of ATP-dependent remodeling complexes, characterized by different core ATPases: SWI/SNF, ISWI, NURD/Mi-2/CHD and INO80. Mutations of SWI/SNF subunits were documented in approximately 20% of human cancers (for example, medulloblastoma, breast cancer), indicating that inactivation of this complex is important in tumor formation (35). PBRM1 encodes the chromatin targeting subunit (BAF180) of the ATP-dependent SWI/SNF chromatin remodeling complex, implicated in proliferation, replication, transcription and DNA repair **(Figure 1)** (36). Truncating mutations in PBRM1 have been found in 88/257 (34%) of ccRCC cases (10). Further studies have shown similar mutation frequencies, making it the second most commonly altered gene in ccRCC, next to VHL (37). However, there is no significant correlation between lack of PBRM1 expression and VHL mutations, and PBRM1 mutations occur at similar rates in tumors with or without VHL mutations (38). Functional *in vitro* assays in ccRCC cell lines with PBRM1 silenced *via* siRNA resulted in a significant increase of proliferation in ACHN and 786-O cell lines (with wild type PBRM1) but not in A704 with a homozygous PBRM1 truncating mutation (10). In turn, reintroduction of PBRM1 into cells induced the cyclin-dependent kinase inhibitor p21 expression and led to reduction in cell proliferation (39). PBRM1 silencing results also in increased colony formation in soft agar and increases cell migration in 786-O, SN12C and TK10 cells, suggesting a tumor suppressive role for PBRM1 in ccRCC (10). Additionally, ccRCCs deficient in PBRM1 are associated with a distinct gene-expression signature enriched for genes implicated in the cytoskeleton and cell motility (40). However, how loss of PBRM1 function affects chromatin modulation patterns and promotes tumorigenesis is unknown.

In a small proportion of ccRCCs, ARID1A (1p35) encoding for different subunit of the SWI/SNF complex (BAF250A) was also found to be mutated **(Figure 1)** (10). In another study, in 16% patients with ccRCC, ARID1A copy number loss was detected - 67% of tumors (n=79) had significantly lower expression of BAF250A than control tissue, and in approximately 70% (n=404) decreased ARID1A mRNA expression was found (41, 42). ARID1A mutations are present at high frequency in other cancers, for example, ovarian clear cell carcinomas (50%), ovarian endometrioid carcinomas (30%), and gastric cancers (29%), and studies have suggested its roles in proliferation, differentiation, and apoptosis (43). The mechanism of ARID1A alterations and their role in ccRCC pathogenesis is still unclear.

Besides chromatin remodeling, histone modifications, controlled by balanced activity of histone modifying enzymes, also play a critical role in maintaining the proper functioning of cells (44). Most common N-terminal tail modifications include acetylation and methylation of lysine or arginine and serine phosphorylation (45). Depending on their type and location, modifications may influence the accessibility of chromatin or can recruit and/or block non-histone effector proteins. Various enzymes are responsible for this dynamic regulation, for example, histone acetyltransferases (HATs) and methyltransferases (HMTs) that add acetyl and methyl groups, respectively, as well as enzymes removing these groups: histone deacethylases (HDACs) and demethylases (HDMs) (46). Altered expression of some of those have been discovered in ccRCC, including SETD2 and MLL2 (methyltransferases) as well as JARID1C/KDM5C and UTX/KDM6A (demethylases) **(Figure 1)**.

SETD2 (SET domain containing protein 2) is mutated in approximately 3% to 8% of ccRCC and its inactivation leads to loss or decrease of trimethylation of lysine 36 of histone H3 (H3K36me3) (10, 11, 47). In addition, a connection has been reported between SETD2 mutations and extensive DNA hypomethylation in ccRCC (16). Similar to VHL and PBRM1, SETD2 is located on chromosome 3p and it was proposed as a novel TSG in ccRCC. A meta-analysis based on 5 different studies suggests SETD2 mutations cooperate with mutations in PBRM1 (48). In addition, Garlinger *et al.* have shown that distinct SETD2 mutations are present in the same tumor, suggesting a high selective pressure to mutate SETD2 (49). How its biallelic inactivation is connected to ccRCC remains unclear. Two studies have linked SETD2 and H3K36me3 to DNA mismatch repair and microsatellite instability of tumors (50, 51). This finding was not confirmed by Kanu *et al.*, who suggest a role for SETD2 in nucleosome reassembly, suppression of replication stress, and the coordination of DNA double-strand breaks (DSBs) repair by homologous recombination (HR) (52). Findings linking SETD2 to HR have been also reported by Carvalho et al., who showed it is required for ATM activation upon formation of DSBs, and for HR repair of DSBs by promoting the formation of RAD51 filaments. SETD2-mutant ccRCC cells displayed impaired DNA damage signaling, decreased cell survival after DNA damage and failure to activate the p53-mediated checkpoint (53). Another methyltransferase frequently mutated in ccRCC, MLL2 (mixed-lineage leukemia protein 2, localized at 12q13.12), directs tri-methylation of histone H3 lysine 4 (11). The role of MLL2 in pathogenesis of ccRCC is currently unknown.

TSG function was also suggested for UTX/KDM6A gene coding for histone demethylase (with 3% mutation frequency in ccRCC) (11, 54). UTX/KDM6A demethylates H3K27me3 linked with repressed chromatin. It associates with MLL2 which also interacts with another H3K4 demethylase JARID1C/KDM5C, found to be frequently deactivated in ccRCC. Loss of JARID1C in 786-O ccRCC cells (VHL ^-/-^) leads to significantly lower H3K4Me3 levels than in VHL^+/+^. JARID1C is proposed to have a tumor suppressor role - its knockdown in 786-O VHL^-/-^ ccRCC cells significantly enhanced tumor growth in a mice xenograft model (55). Taken together, these data implicate deregulation of methylation/demethylation of histone H3 (a major regulator of euchromatin/transcription), as an important and complex phenomenon in ccRCC etiology.

The BRCA1 Associated Protein-1 (BAP1) gene is also often mutated in ccRCC (8–14%) (12, 37, 56). It is located at 3p and codes for a nuclear deubiquitinase targeting H2A, one of the most abundant ubiquitinated proteins in the nucleus, next to H2B **(Figure 1)** (57). BAP1 interacts with Host Cell Factor C1 (HCF-1), which recruits histone-modifying enzymes and serves as a scaffold for chromatin remodeling complexes, promoting the inhibition of cell proliferation (37). Interestingly, BAP1 and PBRM1 mutations are mutually exclusive and loss of either BAP1 or PBRM1 proteins has been observed in approximately 70% of ccRCC cases (37, 56). Moreover, VHL-deficient mice with one active allele of BAP1 exhibited features of human ccRCC, which suggests an important role of BAP1 in the pathogenesis of ccRCC (58).

### Chromatin organization and chromatin accessibility changes

Formaldehyde-assisted isolation of regulatory elements (FAIRE), enables interrogation of chromatin accessibility changes and is based on isolation of nucleosome-depleted regions of DNA, harboring regulatory elements (active transcriptional start sites, transcriptional enhancers, and silencers). Studies using this method showed functional consequences of mutations in genes encoding chromatin regulatory proteins on chromatin organization and transcription in human tumors (59). Buck et al. performed FAIRE on matched pairs of tumor/healthy samples and identified decreased chromatin accessibility at genes previously associated with ccRCC, such as PBRM1, SETD2 and MLL2 (60). Array-based methylation analysis on this same set of tumors revealed that chromatin remodeling can occur in parallel with methylation or independent of it. Recently, Simon et al. used FAIRE to define the chromatin landscape in a cohort of 42 primary ccRCC tumors and 7 matched normal tissues, and studied the possible association of variations in chromatin organization with mutations in SETD2 (61). Changes in chromatin accessibility were identified primarily within actively transcribed genes, and increase in chromatin accessibility was linked to alterations in RNA processing (for example, intron retention and aberrant splicing), affecting ~25% of all expressed genes. Moreover, in tumors lacking H3K36me3 decreased nucleosome occupancy proximal to aberrantly spliced exons was observed. This study links mutations in SETD2 to chromatin accessibility changes and RNA processing defects.

## Epigenetic modifications as markers for ccRCC diagnosis, prognosis, and surveillance

No effective and noninvasive strategy for detection and prognosis of ccRCC has been established to date. ccRCC usually remains asymptomatic until a relatively late stage, therefore early detection, accurate prediction of disease progression and monitoring are critical. Potentially, altered expression of recently reported histone modifiers, might be of clinical relevance **(Table 1)**. ccRCC patients with BAP1 mutations were significantly more likely to present with advanced clinical stage and metastases, and shorter overall survival (56, 62). Similarly, PBRM1 downregulation correlated with advanced tumor stage, low differentiation grade and worse patient outcome while SETD2 mutations correlated with a high relapse rate (38, 56). Moreover, tumors with expression changes of PBRM1 or BAP1, SETD2 and KDM5C were more likely to present with stage III disease or higher (62). Analysis of cancer specific survival (CSS) performed in a large patient cohort of 188 patients and additionally 421 from TCGA, partially confirmed these initial findings (63). BAP1 mutations were associated with worse CSS in both cohorts (MSKCC, p=0.002; TCGA, p=0.002) while SETD2 only in the TCGA cohort (p=0.036). PBRM1 mutations were not correlated with CSS in this study.

Cancer cells display global alterations of DNA methylation, therefore methylation profiling may be implemented in ccRCC biomarker discovery. A specific cancer phenotype designated as the CpG island methylator phenotype (CIMP) was found in ccRCC. It is characterized by DNA hypermethylation of 17 marker genes and by more aggressive tumors, poorer patient outcome, and a higher probability of both, recurrence and disease-related death. ccRCC-CIMP was validated and could be useful for diagnosis and prognostication of the patients (64, 65). A vast amount of aberrantly methylated genes, described in previous paragraphs and exemplified in **Table 1**, may potentially serve as biomarkers (4, 18, 66, 67). However, to predict methylation specificity/sensitivity and thus diagnostic potential, these data require more detailed investigation.

Most studies on both mutation status of histone modifiers and gene methylation were conducted on tissue samples. Fluid based biomarkers for detection, staging and progression monitoring would be more attractive due to easy, non-invasive acquisition. Nevertheless, to date only a limited number of studies aimed at finding specific ccRCC biomarkers in blood or urine has been executed. Methylation-based biomarker candidates found in urine and serum of ccRCC patients, for example, INK4, SFRP1, and SFRP2 were reviewed by Baldewijns *et al*. in 2008 (4). Recently, to our knowledge, only two more reports have been published. RASSF1A, and VHL (detected in serum) as well as KILLIN, and LINE-1 (detected in peripheral blood) have been proposed as predictive biomarkers (68–70). Their association with ccRCC is suggested by significantly higher levels of promoter hypermethylation in ccRCC patients than in patients with benign tumors and healthy controls, respectively. High throughput screening strategies that revealed many new ccRCC biomarker candidates, give hope that in the near future exploration of fluid based epigenetic biomarkers will be intensified.

**Table 1.** Genes involved in epigenetic DNA and chromatin modifications, proposed as potential biomarkers in ccRCC (^a^ genes with methylation frequency above 30%).

	Gene function		Gene name	Locus	Methylation/ mutation frequency	Type of sample	Clinical utility	Type of potential biomarker	Ref.
Promoter methylation a	Wnt/beta-catenin signal transduction pathway	Negative regulation	*DKK1*	10q11	52%	tumor (n=50)	methylation frequency higher in advanced tumor stage	prognostic	(89)
			*DKK2*	4q25	58%	tumor (n=52)	methylation frequency higher in high grade, stage, and size tumors	prognostic	(90)
			*DKK3*	11p15	50%	tumor (n=62)	cancer cell specific methylation	predictive	(91)
		Positive regulation	*SFRP1*	8p11	34%	tumor (n=61)	methylation associated with poor prognosis	prognostic	(27)
			*SFRP2*	4q31	53% 48%	tumor (n=62) serum (n=33)	cancer cell specific methylation methylation frequency higher in high grade and stage tumors	predictive prognostic	(91)
			*SFRP4*	7p14-13	53 %	tumor (n=62)	cancer cell specific methylation	predictive prognostic	(91)
			*SFRP5*	10q24	56% 45%	tumor (n=62) serum (n=33)	cancer cell specific methylation methylation frequency higher in high grade and stage tumors	predictive prognostic	(91)
			*WIF1*	12q14	73%	tumor (n=62)	cancer cell specific methylation	predictive	(91)
	Apoptotic signaling pathway	Pro-apoptotic	*APAF-1*	12q23	41% 41%	tumor (n=90) tumor (n=196)	methylation associated with low overall survival risk of metastatic disease, cancer-related death	prognostic prognostic	(92) (93)
			*DAPK-1*	9q21	64%	tumor (n=196)	frequently methylated in high stage tumors	prognostic	(93)
			*KILLIN*	10q23	95%	tumor (n=20)	cancer cell specific methylation	diagnostic	(68)
	Extracellular matrix structural constituent		*COL1A1*	17q21	65%	tumor (n=20)	frequently methylated in early-stage tumors	prognostic	(94)
			*COL14A1*	8q23	44%	tumor (n=41)	poor prognosis independent of tumor size, stage or grade	prognostic	(27)
			*FBN2*	5q23	40% 52%	tumor (n=199) (n=160)	cancer cell specific methylation	predictive	(95)
	Regulation of transcription		*BNC1*	15q25	46%	tumor (n=61)	poor prognosis independent of tumor size, stage or grade	prognostic	(27)
			*HOXA5*	7p15	51%	tumor (n=62)	methylation frequency higher in high Fuhrman grade tumors	prognostic	(96)
	TSG		*DLEC1*	3p21	31%	tumor (n=81)	methylation frequency higher in more advanced stage tumors	prognostic	(97)
	Inhibitor of TGF signaling		*GREM1*	15q12	63%	tumor (n=147)	high methylation frequency associated with increased tumor size, grade and stage	prognostic	(98)
Chromatin modifiers mutations	SWI/SNF chromatin remodeling complex		*PBRM1*	3p21	29%	tumor (n=185)	mutations associated with advanced tumor stage	prognostic	(61)
	histone H3K4 demethylation		*JARID1C/* *KDM5C*	Xp11	8%	tumor (n=185)	mutations associated with advanced tumor stage	prognostic	(61)
	histone H3K36 trimethylation		*SETD2*	3p21	8% 11% 11%	tumor (n=185) (n=421) (n=106)	mutations associated with worse cancer-specific survival high relapse rate	prognostic prognostic prognostic	(61) (62) (55)
	catalytic subunit of the histone H2A deubiquitinase		*BAP1*	3p21	11% 6% 6% 10%	tumor (n=132) (n=185) (n=188) (n=421)	mutations associated with metastases and advanced tumor stage higher stage & grade tumors; shorter overall survival worse cancer-specific survival	prognostic prognostic prognostic	(99) (61) (62)

## Epigenetic therapies

Studies that highlighted importance of epigenetic modifications in the pathogenesis of ccRCC provided new potential objects for therapeutic intervention. Cancer cells, including ccRCC, are generally characterized by the overexpression of HDACs leading to decreased histone acetylation and consequently silencing of genes involved in the regulation of key cancer pathways (71, 72). Several studies proved the efficacy of some HDAC inhibitors in reducing tumor growth in cancer patients in phase I and II clinical trials (72–74). Currently, HDACs are intensively explored as targets of ccRCC therapy (67, 75). Monotherapies such as, with panobinostat, did not bring satisfactory results to date. A phase II study enrolled 20 patients with metastatic refractory ccRCC, previously treated with mTOR inhibitor(s). In the first evaluation, five patients showed stable disease and three patients experienced progression. Treatment was generally well tolerated but the median progression-free survival was limited to 17 months. Hence, panobinostat is recommended only in combination with other anticancer drugs (76). Also depsipeptide, tested in 29 patients with metastatic RCC (ccRCC n=25) in a phase II study, did not show satisfactory results as a monotherapy. The overall treatment response rate was 7%, in addition severe side effects like fatigue, nausea, vomiting, anemia were observed (77).

Combined treatment approaches with HDAC inhibitors seem to be more effective than monotherapy. In models of RCC, the HDAC inhibitor vorinostat improved the anticancer activity of temsirolimus (78). Reduced cell viability, clonogenic survival and increased cell death was observed in RCC cell lines (86-O, A498, 769-P, Caki-1, Caki-2, SW839, ACHN, G401 and SK-NEP-1) in response to combined treatment. In xenografts of RCC cell lines (786-O and Caki-1), vorinostat inhibited tumor cell proliferation, induced apoptosis and impaired angiogenesis, through a decrease in HIF-2a expression and vessel density. *In vitro* and *in vivo* studies have also shown that a combination of retinoic acid and HDAC inhibitor trichostatin A is more efficient than each drug alone (79). The combined therapy enhanced the retinoic acid pathway signaling, leading to a reduction of proliferation of human RCC cells lines (SK-RC-39 and SK-RC-45), inhibition of tumor model growth (SK-RC-39) and increased apoptosis. In combination with retinoids, also MS-275, a benzamine derivative HDAC inhibitor, showed a better inhibitory effect on tumor growth *in vivo*. This effect persisted after treatment withdrawal, and after continuous treatment in animals RCC1.18 tumor progression was not observed (80). Interestingly, an induction of retinoic acid receptor beta was observed during treatment, suggesting HDAC inhibitors might revert retinoid resistance.

There are also attempts to develop drugs selectively targeting other enzymes involved in epigenetic modulation, especially histone methyltransferases or histone demethylases. There are a few methyltransferase inhibitors showing promising results in cancer models (75, 81). In ccRCC, the S-adenosylhomocysteine hydrolase inhibitor, 3-deazaneplanocin A (DZNep), depletes cellular levels of the enhancer of zeste homologue 2 (EZH2). EZH2 is a catalytic subunit of the polycomb repressive complex 2 (PRC2), a histone methyltransferase that catalyzes tri-methylation of lysine 27 on histone 3 (82). DZNep reduces H3K27 trimethylation levels, additionally, RCC cells exposed to DZNep showed a significant decrease of cell migration and invasion in vitro, as well as inhibition of tumor growth, and prolonged survival in the in vivo mice model.

In a recent report published by Adelaiye *et al.*, resistance to sunitinib was studied in mice bearing two different patient-derived ccRCC xenografts (83). Increasing the drug dose led to partial overcome of initial sunitinib-induced resistance, suggesting its association with epigenetic changes such as overexpression of the methyltransferase EZH2 and modulation of histone marks. Moreover, specific EZH2 inhibition resulted in increased in vitro anti-tumor effect of sunitinib. These promising results indicate that high throughput screening strategies could be used to identify further drug-candidates.

## Perspectives

Availability of high-throughput methods have facilitated investigation of epigenetic modifications in general. The Roadmap Epigenomics Program recently published mapped epigenomes of 111 types of primary human healthy cells and tissues, providing valuable reference epigenome maps (84), moreover many epigenome-wide association studies (EWASs) initiated in various diseases are currently intensively conducted (85). Epigenetic studies have also widely broadened our understanding of the biology of ccRCC, providing evidence of various DNA mutation and methylation events, chromatin alterations and changes of DNA accessibility, and altogether suggesting that epigenetic alterations are connected to ccRCC pathogenesis/progression and require further detailed examination. A number of new large-scale projects seeking RCC biomarkers are currently ongoing, for example, CAGEKID, “Biomarker pipeline” (NIH), EuroTARGET or the PREDICT consortium (66, 86–89). These studies are expected to identify and characterize novel candidate biomarkers for ccRCC detection, staging and monitoring.
